# Expression of proposed methionine transporters along the gastrointestinal tract of pigs and their regulation by dietary methionine sources

**DOI:** 10.1186/s12263-021-00694-4

**Published:** 2021-09-06

**Authors:** Stella Romanet, Jörg R. Aschenbach, Robert Pieper, Jürgen Zentek, John K. Htoo, Rose A. Whelan, Lucia Mastrototaro

**Affiliations:** 1 Institute of Veterinary Physiology, Freie Universität Berlin, Oertzenweg 19b, 14163 Berlin, Germany; 2 Institute of Animal Nutrition, Freie Universität Berlin, Berlin, Germany; 3 Evonik Operations GmbH, Animal Nutrition Services, Hanau-Wolfgang, Germany

**Keywords:** Longitudinal heterogeneity of gene expression, Intestine, Methionine transport, qRT-PCR, Stomach, Western blot

## Abstract

**Background:**

Given the key role of methionine (Met) in biological processes like protein translation, methylation, and antioxidant defense, inadequate Met supply can limit performance. This study investigated the effect of different dietary Met sources on the expression profile of various Met transporters along the gastrointestinal tract (GIT) of pigs.

**Methods:**

A total of 27 pigs received a diet supplemented with 0.21% DL-Met, 0.21% L-Met, or 0.31% DL-2-hydroxy-4-(methylthio)butanoic acid (DL-HMTBA). Changes in mRNA expression of *B*^*0*^*AT1*, *ATB*^*0,+*^, *rBAT*, *ASCT2*, *IMINO*, *LAT4*, *y*^*+*^*LAT1*, *LAT2*, and *SNAT2* were evaluated in the oral mucosa, cardia, fundus, pylorus, duodenum, proximal jejunum, middle jejunum, ileum, cecum, proximal colon, and distal colon, complemented by protein expression analysis of B^0^AT1, ASCT2, LAT2, and LAT4.

**Results:**

Expression of all investigated transcripts differed significantly along the GIT. *B*^*0*^*AT1*, *rBAT*, *y*^*+*^*LAT1*, *LAT2*, and *LAT4* showed strongest mRNA expression in small intestinal segments. *ASCT2, IMINO*, and *SNAT2* were similarly expressed along the small and large intestines but expression differed in the oral mucosa and stomach. *ATB*^*0,+*^ showed highest mRNA expression in large intestinal tissues, cardia, and pylorus. In pigs fed DL-Met, mRNA expression of *ASCT2* was higher than in pigs fed DL-HMTBA in small intestinal tissues and mRNA expression of *IMINO* was lower than in pigs fed L-Met in large intestinal tissues. Dietary DL-HMTBA induced a stronger mRNA expression of basolateral uptake systems either in the small (*LAT2*) or large (*y*^*+*^*LAT1*) intestine. Protein expression of B^0^AT1 was higher in the middle jejunum and ileum in pigs fed DL-Met when compared with the other Met supplements. LAT4 expression was higher in pigs fed DL-HMTBA when compared with DL-Met (small intestine) and L-Met (small intestine, oral mucosa, and stomach).

**Conclusion:**

A high expression of several Met transporters in small intestinal segments underlines the primary role of these segments in amino acid absorption; however, some Met transporters show high transcript and protein levels also in large intestine, oral mucosa, and stomach. A diet containing DL-Met has potential to increase apical Met transport in the small intestine, whereas a diet containing DL-HMTBA has potential to increase basolateral Met transport in the small intestine and, partly, other gastrointestinal tissues.

## Introduction

Methionine (Met) is an essential sulfur amino acid (AA), which must be provided by the diet because it cannot be synthesized *de novo* by the body [1]. Methionine plays several essential roles in cellular metabolism. It is a proteinogenic AA with special importance for the initiation of protein translation [2], a sulfur donor necessary to generate other sulfur-containing AA (cysteine and cystine), a main donor of methyl groups [3] and, finally, it influences the cellular redox state [4].

The optimum AA ratios in food or feed is a key element to ensure coverage of AA requirements with a minimum of protein intake [5, 6]. In animal production, reducing dietary protein levels is not only vital to reduce N excretion through urine and feces but also to lower greenhouse gas emissions [7, 8]. Additionally, the reduction of protein levels in the feed of livestock is a cost-reducing strategy [5, 9]. Therefore, providing adequate dietary methionine supply, as a main limiting essential AA in low crude protein diets, is crucial to ensure optimal growth and health with additional ecologic and economic benefits. Whereas humans rely primarily on naturally occurring L-Met from food materials, pig and poultry diets are often supplemented with L-Met, DL-Met, or a hydroxyl analogue DL-2-hydroxy-4-(methylthio)butanoic acid (DL-HMTBA) [10, 11]. Not only are the metabolism and utilization different for these Met sources; they also differ in their absorption mechanisms. Because HMTBA is a precursor without an amino group, it is not absorbed by AA transporters, but rather by sodium-dependent and sodium-independent monocarboxylate transporters, one prominent candidate of the latter being MCT1 [12, 13]. By sharp contrast, Met is mainly taken up via carrier-mediated systems that differ in their specificity for L- and D-isomers [11, 14]. The current knowledge on gastrointestinal Met transporters has been summarized by Mastrototaro et al. [14]. According to this proposed model, the apical transport systems are mainly Na^+^-dependent (B^0^AT1, ATB^0,+^, ASCT2, IMINO) but are complemented by b^0,+^AT which is Na^+^-independent. b^0,+^ requires the heavy chain rBAT for membrane targeting; hence, expression of rBAT is considered critical for b^0,+^/rBAT heterodimer function. Consequently, previous studies mostly analyzed the expression of subunit rBAT in preference over subunit b^0,+^ [15–17]. On the basolateral side, the main transport system for Met efflux is represented by the Na^+^-independent system L (LAT1, LAT2, and LAT4; where L stands for large neutral AA). Interestingly, the exact localization of LAT1 seems to be species-specific as it was located basolaterally in the chicken intestine and porcine kidney but apically in human intestine [12, 18]. System L is complemented by the Na^+^-dependent transporters SNAT2 and y^+^LAT1. SNAT2 takes up AA from blood in interdigestive phases, and y^+^LAT1 mediates the Na^+^-dependent influx of neutral AA (like Met) against an efflux of cationic AA [19–21].

The present study aimed to determine whether the supplementation of different dietary Met sources would modulate the distribution and the expression profile of presumed Met transporters along the porcine GIT. In a previous study on tissues from the same animals, we had identified increased transport of L- and partly D-Met in different small intestinal segments (duodenum, middle jejunum, and ileum) after feeding a DL-Met–containing diet [22].

## Experimental procedures

### Animals and diets

Diets and animal handling procedures have been described previously [22]. Briefly, 27 pigs (castrated male, Danbred x Piétrain) were used with an initial bodyweight of ~ 25 kg at ~ 10 weeks of age to assure the establishment of stable gastrointestinal health after weaning. Pigs were fed a basal diet deficient in standardized ileal digestible (SID) Met + Cys (0.46%) but adequate for all other AA. To meet Met + Cys requirements, the basal diet was supplemented with either 0.21% DL-Met (MetAMINO; Evonik Nutrition & Care GmbH, Essen, Germany; *N* = 9), 0.21% L-Met (Evonik Nutrition & Care GmbH, Essen, Germany; *N* = 9) or 0.31% DL-HMTBA (Novus International, Inc., Saint Charles, MO; *N* = 9). The higher dietary concentration of DL-HMTBA was used to account for its lower bioefficacy (approximately 70% compared with L-Met) [10]. Diets were provided for at least 10 days, after which pigs were euthanized for harvesting tissues. Dietary treatments were blinded to the research investigators until gene expression data was summarized.

### Tissue preparation

After euthanasia, tissue samples of all pigs were recovered from a total of 11 regions of the GIT for molecular analyses (quantitative real-time PCR and western blot) of Met transporter expression. Sections included four extraintestinal regions (oral mucosa as well as cardia, fundus, and pylorus of the stomach), four small intestinal regions (duodenum, proximal jejunum, middle jejunum, and ileum), and three large intestinal regions (cecum, proximal colon, and distal colon). The study targeted at selective quantification of epithelial transporters. Therefore, the tunica muscularis externa (longitudinal and circular muscle layers) was mechanically removed before collecting gastric and intestinal samples. Oral mucosa was harvested by perpendicular cutting of small tissue chips from the mucosal surface.

Tissues for transcript expression analysis were immersed in RNA*later*® (Sigma Aldrich, St Louis, MO, USA), stored at + 4 °C overnight and at – 20 °C thereafter until RNA isolation. Tissues for protein analysis were snap-frozen in liquid nitrogen and then stored at – 80 °C until analysis.

### Gene expression analysis

Total RNA was extracted from the tissues using a commercial kit including a DNAse digestion step (Nucleospin RNA, Macherey & Nagel, Düren, Germany). Afterwards, all RNA samples were evaluated for quantity and purity using a lab-on-a-chip technique (RNA 6000 Nano Kit, Agilent, Waldbronn, Germany). Only samples with an RNA integrity number (RIN) > 6.5 were used for cDNA synthesis. Reverse transcription was performed with 1000 ng of RNA using iScript® cDNA synthesis kit (Bio-Rad Laboratories, Munich, Germany) according to the manufacturer’s instructions; reactions were then diluted to a final concentration of 5 ng/μL.

Changes in the relative expression of the Met transporters *B*^*0*^*AT1*, *ATB*^*0,+*^, *rBAT*, *ASCT2*, *IMINO*, *LAT2, LAT4*, *y*^*+*^*LAT1*, and *SNAT2* were evaluated by qRT-PCR using intron-flanking or exon-spanning primers and double quenched probes synthetized by Eurofins MWG Operon (Ebersberg, Germany; for primer and probe sequences, see Table 1).

**Table 1:** Primer and probe sequences for the Met transporters and reference genes

Gene	Accession number	Primer	Sequence	Probe sequence
*B*^*0*^*AT1* *(SLC6A19)*	XM_003359855.4	Fwd	CTTCATCTTCACCCTGAACTC	CCCCTGCTCATCATCGCCTTCTTCGAGATGT
		Rev	GATGTCGCTGTTGAACCTG	
*ATB*^*0+*^ *(SLC6A14)*	NM_001348402.1	Fwd	CTGTGGCTTGGGGTGGTTTA	CCAACTCCCAGGTGGGCCAT
		Rev	AACCAAGCAGCAACCCAAAG	
*rBAT* *(SLC3A1)*	NM_001123042.1	Fwd	CAATGCAGTGGGACAACAG	TCCAAAAGACCCAGCCCAAATCAGCA
		Rev	GGCGTGAAGCAAACTTAATTC	
*ASCT2* *(SLC1A5)*	XM_003127238.4	Fwd	CGATTCGTTCCTGGATCTTG	CTCCAACCTGGTGTCTGCAGCCTT
		Rev	TAGGACGTCGCGTATGAG	
*IMINO* *(SLC6A20)*	XM_003358406.4	Fwd	TCGTGTCCCTCATCAACAG	ACCTCCATCTTTGCCAGTGTCGTCACCTT
		Rev	AGGAAGCCATCTTCAAGGTC	
*LAT4* *(SLC43A2)*	XM_003358191.3	Fwd	CAGATCCAGAAGATCACCAAC	TGACCTGCCTCATTCCCAACCTGC
		Rev	TGAAGGAGAGAATCTGTAGGG	
*y*^*+*^*LAT1* *(SLC7A7)*	NM_001110421.1	Fwd	CTCTGCTGTTCAATGGTCTC	GGCGCTGATCTACTTGTGCGTGGA
		Rev	ATAGAGCTGACCCACGATAG	
*LAT2* *(SLC7A8)*	XM_003128550.5	Fwd	ACTACCTCTTCTATGGCATCAC	CGGACAGATAGTTCTTCGCTGGAAGAAGCCTAA
		Rev	GCAAGTAGATGATGGGGAACAG	
*SNAT2* *(SLC38A2)*	XM_003126626.5	Fwd	TTCATTCTTCCATCTGCCTTC	GTTAAGTGGCATAGGGGTGATGACCGGA
		Rev	GGGCATTGTGTACCCAATC	
*ACTB*	XM_021086047.1	Fwd	GACATCAAGGAGAAGCTGTG	CTGGACTTCGAGCAGGAGATGGCC
		Rev	CGTTGCCGATGGTGATG	
*GAPDH*	XM_021091114.1	Fwd	CAAGAAGGTGGTGAAGCAG	TGAGGACCAGGTTGTGTCCTGTGACTTCAA
		Rev	GCATCAAAAGTGGAAGAGTG	
*YWHAZ*	XM_005662949.2	Fwd	AAGAGTCATACAAAGACAGCAC	ATCGGATACCCAAGGAGATGAAGCTGAA
		Rev	ATTTTCCCCTCCTTCTCCTG	

The qRT-PCR experiments used a 40-cycle two-step PCR protocol (20 s at 60 °C and 1 s at 95 °C) and were performed in a thermocycler (ViiA7, Applied Biosystems/Life Technologies, Foster City, CA, USA) with 4.5 μL of cDNA and three replicates per reaction. iTaq® Universal Probes Supermix (Bio-Rad Laboratories) in combination with the specific primers and probes was used as master mix in assay volumes of 10 μL. Thresholds were automatically calculated by the cycler software. Amplicons were validated by sequencing.

Glyceraldehyde 3-phosphate dehydrogenase (*GAPDH*), tyrosine 3-monooxygenase/tryptophan 5-monooxygenase activation protein zeta (*YWHAZ*), and *β-actin* were tested for stable expression with geNorm, and all three were suitable and used as nonregulated reference genes. An inter-run calibrator (IRC), composed of a pool of 21 cDNA, was present on each plate and afterwards used as calibrator. The double delta C_t_ analysis was performed to analyze qRT-PCR data; so after normalization of Ct values with the reference genes, the normalized results were scaled to the calibrator to obtain the expression fold change of each sample relative to the IRC. Calibrated normalized relative quantities (CNRQ values) were used for statistical analysis.

### Protein analysis

Proteins were isolated from ~ 200 mg of frozen tissue samples homogenized in 500 μL of RIPA buffer containing 50 mM Tris, 150 mM NaCl, 1% Triton X-100, 0.1% SDS, 2 mM EDTA, and 5 μL of Protease Inhibitor Mix G (Serva, Heidelberg, Germany). The samples were incubated on ice for 90 min and briefly shaken at 20,000 rpm for 1.5 min every 20 min by using a Mixer Mill (Retsch MM200, Hahn, Germany). Centrifugation (14,000 rpm, 4 °C, 30 min) was performed to a pellet-insolubilized material. The concentration of total extracted proteins was determined using the Pierce® 660-nm Protein Assay (ThermoFisher Scientific, Waltham, MA, USA). An aliquot of 15 μg total protein was resolved on an 8% SDS-polyacrylamide gel for LAT2, LAT4, and B^0^AT1. The proteins for ASCT2 were loaded on a 10% TGX Stain-Free gel™ (Bio-Rad Laboratories). An IRC was loaded as reference sample on each gel.

**Fig. 1:**
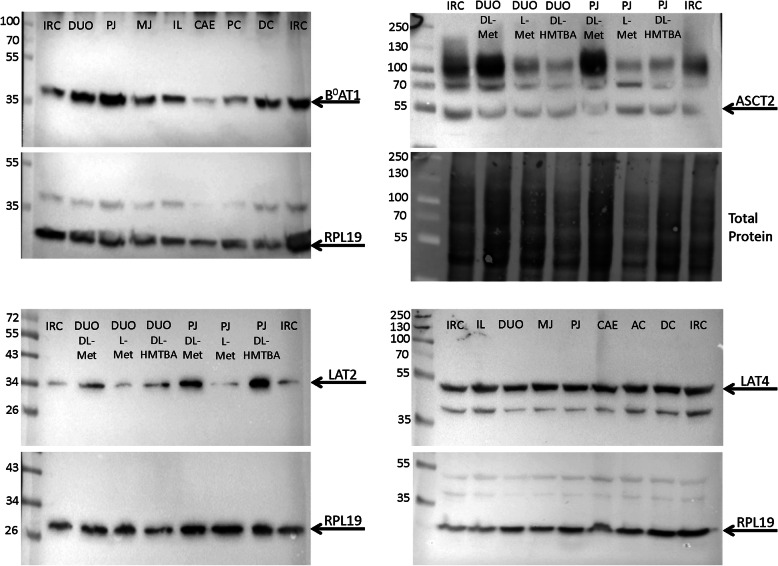
Western blots of B^0^AT1, ASCT2, LAT2, and LAT4 protein. Blots are representative of a total of 188 western blots evaluated for this study. Blots for B^0^AT1, LAT2, and LAT4 were conventional western blots where specific signals were quantified relative to RPL19 as loading control. For ASCT2, stain-free technology was used and ASCT2 protein intensity was quantified relative to total protein. Tissues were grouped per animal on blots for B^0^AT1 and LAT4, whereas tissues from the three feeding groups were compared on blots for LAT2 and ASCT2. The left lane in each blot shows the molecular weight marker. *IRC*, inter-run calibrator; *DUO*, duodenum; *PJ*, proximal jejunum; *MJ*, middle jejunum; *IL*, ileum; *CAE*, caecum; *PC*, proximal colon; *DC*, distal colon

Following electrophoresis, the proteins were transferred to a polyvinylidene difluoride (PVDF) membrane (Bio-Rad Laboratories Inc.), which was blocked with 5% dry milk in TBS + Tween (TBST, 50 mM Tris, 150 mM NaCl, 0.01% Tween-20, pH 7.6) for 2 h at room temperature. After blocking, immunoblotting was performed overnight at 4 °C with a primary rabbit antibody directed against B^0^AT1 (SLC6A19, 1:1000; ABIN567031, Abnova Corp., Taipeh, Taiwan), mouse antibody against ASCT2 (SLC1A5, 1:1000; NBP1-89327 Novus biologicals, Littleton, CO), rabbit antibody against LAT2 (SLC7A8, 1:1000; ABIN2781629, Aviva Systems Biology, San Diego, CA, USA) or mouse antibody against LAT4 (SLC43A2, 1:1000; ABIN2781629 Abgent, San Diego, CA, USA). Primary mouse antibody specific for RPL19 (1:1500; Santa Cruz Biotechnology, Dallas, TX, USA) was used to quantify RPL19 as reference protein to control for loading efficiency for LAT2, LAT4, and B^0^AT1. For ASCT2, total protein on the gel was quantified by the stain-free technology according to the manufacturer’s instruction and used to correct for loading efficiency. After overnight incubation with the primary antibodies, the membranes were incubated with the respective horseradish peroxidase (HRP)-conjugated secondary antibodies (anti-mouse, 1:1000; anti-rabbit, 1:2500; both from Cell Signaling Technology, Frankfurt, Germany). Proteins were visualized by use of the Clarity^TM^ Western ECL Substrate (Bio-Rad Laboratories) and the Bio-Rad ChemiDoc^TM^ MP Imaging System in combination with the software ImageLab 5.0 (Bio-Rad Laboratories), which allowed a densitometric analysis and a normalization of each band to the blackness of the respective lane (normalized intensities, NI). IRC was always assumed to have NI = 1; thus any other value represented the relative expression of the respective sample compared with IRC. Representative western blots are shown in Fig. 1.

### Statistical analysis

The comparisons of mRNA or protein levels between all regions irrespective of the factor diet (Tables 2) were performed with IBM SPSS Statistics Version 26 (IBM, Armonk, NY, USA), using ANCOVA (Analysis of covariance) followed by post hoc Dunn’s method (all pairwise multiple comparison), accounting for the covariate diet. The effect of diet on the expression of genes and proteins in comparable sets of regions (extraintestinal, small intestinal, and large intestinal regions) were subsequently compared by two-factor ANOVA with post hoc Student-Newman-Keuls’ test (all pairwise multiple comparison) using the software SigmaPlot 11.0 (Systat Software, GmbH, Erkrath, Germany). Fixed factors were “diet” (DL-Met, L-Met, and DL-HMTBA) and “tissue”. Results are given as means ± SEM. Differences of *P* < 0.05 were considered significant; trends are discussed if 0.05 ≤ *P* < 0.1.

**Table 2:** mRNA expression of Met transporters among different intestinal and extraintestinal regions irrespective of the factor diet

Tissue	***B***^***0***^***AT1***	***ATB***^***0+***^	***rBAT***	***ASCT2***	***IMINO***	***y***^***+***^***LAT1***	***LAT2***	***LAT4***	***SNAT2***
**Oral mucosa**	0.014 ± 0.006^d^	0.48 ± 0.12^cd^	0.019 ± 0.008^d^	0.57 ± 0.15^b^	0.065 ± 0.023^b^	0.072 ± 0.016^e^	0.14 ± 0.02^d^	0.10 ± 0.02^d^	0.69 ± 0.12^c^
**Cardia**	0.023 ± 0.008^d^	1.76 ± 0.25^ac^	0.20 ± 0.03^d^	0.74 ± 0.11^b^	0.88 ± 0.09^a^	0.37 ± 0.03^ce^	0.49 ± 0.05^bcd^	0.33 ± 0.07^d^	0.92 ± 0.11^bc^
**Fundus**	0.034 ± 0.011^d^	0.040 ± 0.010^d^	0.025 ± 0.005^d^	1.74 ± 0.29^a^	0.26 ± 0.04^b^	1.51 ± 0.19^ab^	2.23 ± 0.28^a^	0.59 ± 0.07^d^	1.59 ± 0.18^a^
**Pylorus**	0.022 ± 0.005^d^	1.09 ± 0.27^bd^	0.025 ± 0.004^d^	1.82 ± 0.30^a^	0.75 ± 0.06^a^	0.40 ± 0.04^ce^	0.65 ± 0.08^bcd^	0.23 ± 0.05^d^	1.37 ± 0.13^ab^
**Duodenum**	0.55 ± 0.08^c^	1.04 ± 0.61^bd^	2.34 ± 0.38^ab^	0.80 ± 0.13^b^	0.85 ± 0.06^a^	1.06 ± 0.13^bc^	0.64 ± 0.07^b^	1.24 ± 0.19^b^	0.70 ± 0.07^c^
**Proximal jejunum**	0.91 ± 0.11^bc^	0.43 ± 0.08^cd^	3.13 ± 0.87^a^	0.71 ±0.12^b^	0.91 ± 0.11^a^	1.12 ± 0.20^b^	0.64 ± 0.09^bcd^	1.92 ± 0.24^a^	0.65 ± 0.06^c^
**Middle jejunum**	1.73 ± 0.21^a^	0.15 ± 0.06^d^	1.31 ± 0.86^c^	0.63 ± 0.11^b^	0.96 ± 0.08^a^	2.11 ± 0.36^a^	2.02 ± 0.33^a^	1.81 ± 0.32^ab^	0.62 ± 0.06^c^
**Ileum**	1.13 ± 0.12^b^	0.21 ± 0.03^d^	1.56 ± 0.25^bc^	0.52 ± 0.08^b^	1.04 ± 0.13^a^	0.91 ± 0.16^bcd^	0.63 ± 0.07^bc^	1.32 ± 0.15^ab^	0.85 ± 0.17^bc^
**Cecum**	0.010 ± 0.002^d^	1.60 ± 0.35^ac^	0.17 ± 0.04^d^	0.83 ± 0.13^b^	0.79 ± 0.09^a^	0.26 ± 0.02^de^	0.28 ± 0.02^cd^	0.28 ± 0.03^d^	0.97 ± 0.11^bc^
**Proximal colon**	0.026 ± 0.008^d^	2.34 ± 0.30^ab^	0.093 ± 0.020^d^	0.81 ± 0.13^b^	0.95 ± 0.16^a^	0.28 ± 0.02^de^	0.43 ± 0.05^bcd^	0.27 ± 0.04^d^	1.05 ± 0.14^ac^
**Distal colon**	0.013 ± 0.003^d^	2.82 ± 0.60^a^	0.10 ± 0.02^d^	1.17 ± 0.23^ab^	1.04 ± 0.19^a^	0.33 ± 0.03^de^	0.55 ± 0.08^bcd^	0.32 ± 0.04^d^	0.84 ± 0.13^bc^
***P*value**	< 0.001	< 0.001	< 0.001	< 0.001	< 0.001	< 0.001	< 0.001	< 0.001	< 0.001

^a–e^Gene expressions within one column are different at *P* < 0.05 if they do not share a common letter.

Graphs were plotted with SigmaPlot 11.0. *P* values for main factors and their interactions are listed in each graph. For clarity, however, only relevant *P* values < 0.1 are shown. If *P* values are not listed in a graph, they are ≥ 0.1.

## Results

### Effects of tissue and diet on mRNA expression

The regional distribution of Met transporters irrespective of the provided diet is shown in Table 2. Relative expression data differed among the gastrointestinal sections for all investigated transcripts (*P* < 0.001). Of note, *B*^*0*^*AT1*, *rBAT*, and *LAT4* had higher mRNA expression in the small intestinal segments compared with all other segments. *B*^*0*^*AT1* had highest CNRQ values in the middle jejunum, *rBAT* in the duodenum and proximal jejunum, and *LAT4* in the proximal and middle jejuna. *y*^*+*^*LAT1* showed highest CNRQ values in the middle jejunum and fundus, with intermediate CNRQ values in the duodenum, proximal jejunum, and ileum. *ASCT2*, *IMINO*, and *LAT2* had similar CNRQ values in most segments, except for a high CNRQ of *ASCT2* in the gastric fundus, gastric pylorus and distal colon, a comparatively low CNRQ of *IMINO* in the oral mucosa and gastric fundus, and a high CNRQ of *LAT2* in the gastric fundus and middle jejunum. *SNAT2* mRNA was highly expressed in the gastric fundus and pylorus. Expression of *ATB*^*0,+*^ appeared dominant in the large intestine with highest CNRQ values measured in the proximal and distal colons; intermediate values in the cecum, gastric cardia, gastric pylorus, and duodenum; and low expression in the jejunum, ileum, and gastric fundus. Oral mucosa was among the tissues with the weakest expression levels for all transporters tested (*P* < 0.05; Table 2).

As different gastrointestinal regions have different digestive functions, three functional subgroups with comparable functions were created to analyze the effect of diet on transporter expression: extraintestinal tissues (oral mucosa and the three gastric regions pylorus, cardia, and fundus), small intestinal tissues (duodenum, proximal jejunum, middle jejunum, and ileum), and large intestinal tissues (proximal colon, distal colon, and cecum).

**Fig. 2:**
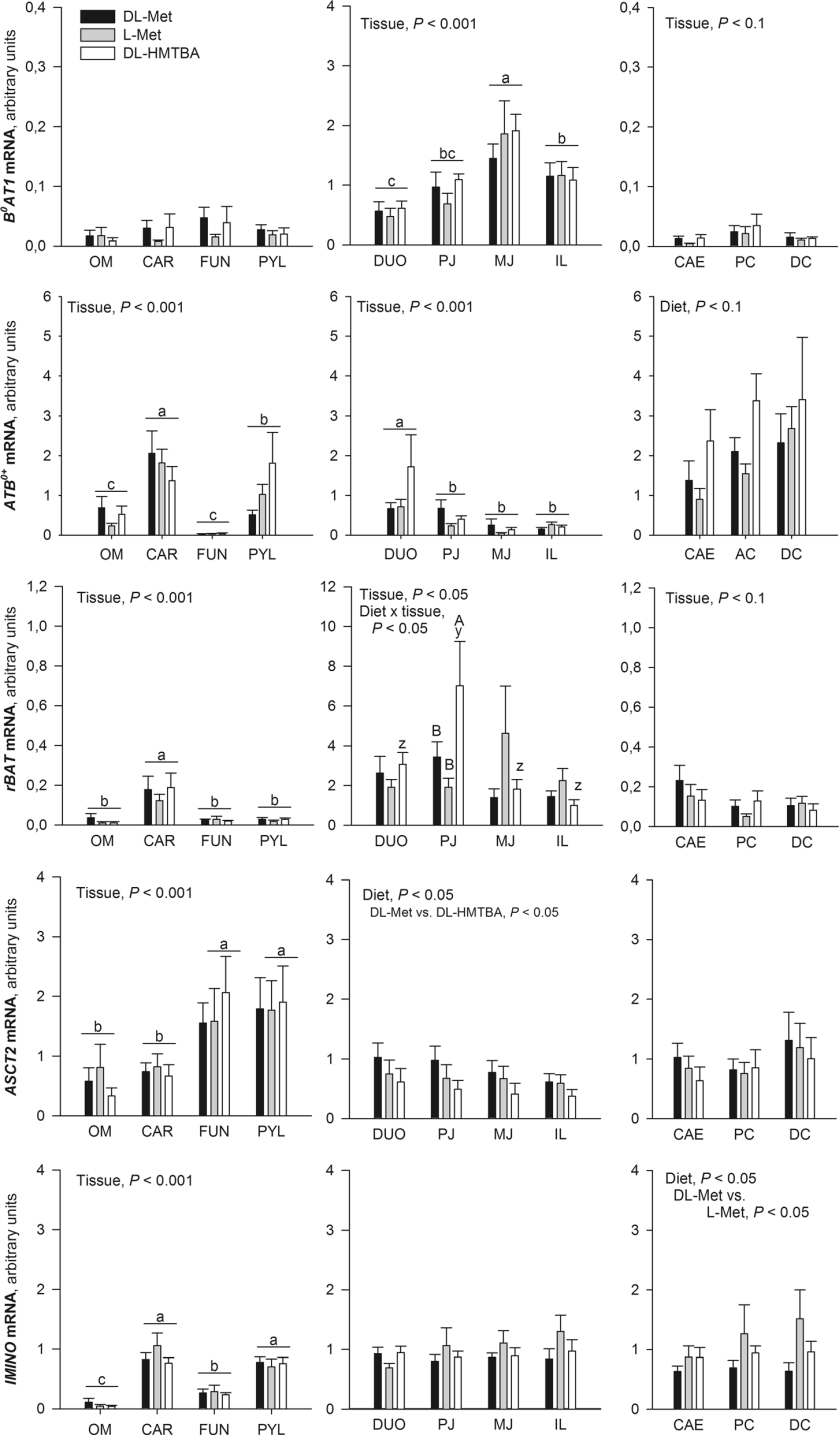
Analysis of Met transporter expression with proposed apical localization along the gastrointestinal tract using qRT-PCR. Data was compared with 2-way-ANOVA for the factors “Tissue”, “Diet”, and their interaction “Tissue x Diet”. Significant factor effects are mentioned in each graph. If column groups or columns do not share a common small letter within one graph, their expression values are either different irrespective of diet^(a–c)^ or within a given diet^(y,z)^ (*P* < 0.05). ^A,B^Different capital letters indicate diet effects within a given tissue. *OM*, oral mucosa; *CAR*, cardia; *FUN*, fundus; *PYL*, pylorus; *DUO*, duodenum; *PJ*, proximal jejunum; *MJ*, middle jejunum; *IL*, ileum; *CAE*, cecum; *PC*, proximal colon; *DC*, distal colon

**Fig. 3:**
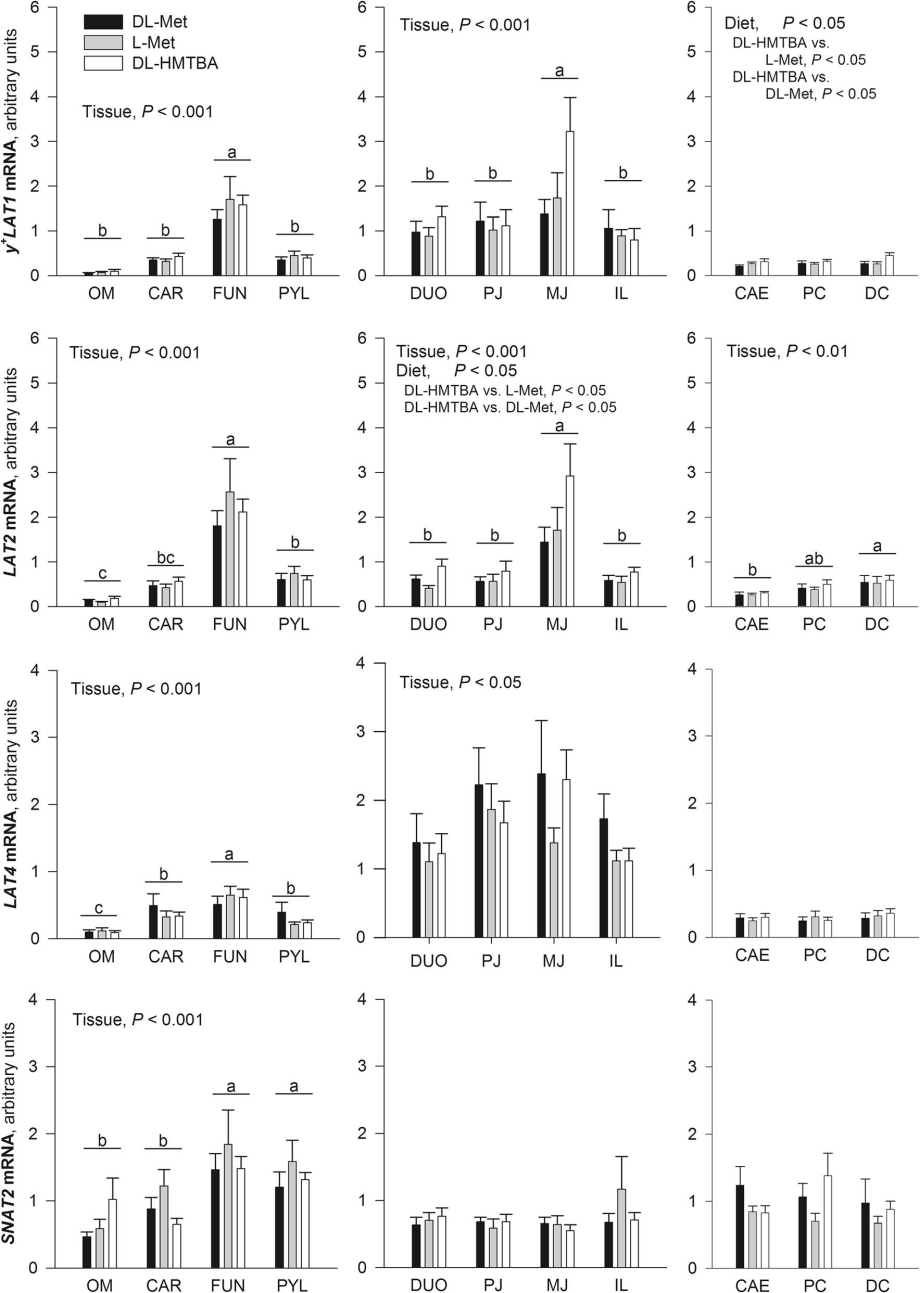
Analysis of Met transporter expression with proposed basolateral localization along the gastrointestinal tract using qRT-PCR. Data was compared with 2-way-ANOVA for the factors “Tissue”, “Diet”, and their interaction “Tissue x Diet”. Significant factor effects are mentioned in each graph. ^a–c^ Expression values among tissues within one graph (irrespective of diet) are different if they do not share a common letter (*P* < 0.05). For *LAT4*, multiple comparison could not identify differences in the small intestine despite of a significant effect of tissue. *OM*, oral mucosa; *CAR*, cardia; *FUN*, fundus; *PYL*, pylorus; *DUO*, duodenum; *PJ*, proximal jejunum; *MJ*, middle jejunum; *IL*, ileum; *CAE*, cecum; *PC*, proximal colon; *DC*, distal colon

Transport systems with supposedly apical and basolateral locations are shown in Figs. 2 and 3, respectively. The factor diet had no effect on any transporter in the extraintestinal tissues. In selected intestinal tissues, the expression of *rBAT*, *ASCT2*, *IMINO*, *y*^*+*^*LAT1,* and *LAT2* was affected by the factor diet. A diet containing DL-Met increased *ASCT2* gene expression across all small intestinal tissues compared with a diet containing DL-HMTBA (*P* < 0.05; Fig. 2). A diet containing L-Met induced a stronger expression of *IMINO* across large intestinal tissues compared with DL-Met (*P* < 0.05; Fig. 2). The diet containing DL-HMTBA increased the expression of the basolateral exchange systems *LAT2* in the small and *y*^*+*^*LAT1* in the large intestines (*P* < 0.05; Fig. 3). It also caused an increase in the expression of *rBAT* selectively in the proximal jejunum (*P* < 0.05) as evidenced by a diet × tissue interaction (*P* < 0.05; Fig. 2).

The two-factorial statistical evaluation of regions further supported the differences among tissues that had already been identified by ANCOVA (cf. Table 2). Additional findings were that cardia had a higher expression of *ATB*^*0,+*^ and *rBAT* than other extraintestinal regions; in the small intestine, the duodenum had highest expression of *ATB*^*0,+*^, distal colon had higher expression of *LAT2* than caecum, and that expression patterns of *LAT2* and *LAT4* showed clear regional differences in extraintestinal tissues (Fig. 3).

### Effects of tissue and diet on protein expression

Selected transporters with assumed high relevance for apical Met uptake (B^0^AT1 and ASCT2) or Met basolateral efflux (LAT2 and LAT4) were further investigated on the protein level by western blot. As for mRNA, data from the three feeding groups were initially compared across all tissues by ANCOVA accounting for diet as covariate. Protein expression levels of all investigated proteins were significantly different along the GIT (*P* < 0.001; Table 3.

**Table 3:** Protein expression data of selected methionine transporters among different intestinal and extraintestinal regions irrespective of the factor diet

Tissue	B^**0**^AT1	ASCT2	LAT2	LAT4
**Oral mucosa**	0.60 ± 0.11^cd^	1.70 ± 0.26^ac^	4.13 ± 0.44^b^	7.50 ± 0.70^a^
**Cardia**	1.41 ± 0.25^ab^	0.78 ± 0.14^c^	7.45 ± 1.70^a^	0.98 ± 0.23^bc^
**Fundus**	0.26 ± 0.05^d^	0.62 ± 0.16^c^	2.89 ± 0.64^bc^	0.11 ± 0.03^c^
**Pylorus**	0.67 ± 0.12^cd^	0.77 ± 0.12^c^	0.79 ± 0.14^d^	1.00 ± 0.29^bc^
**Duodenum**	0.79 ± 0.12^bd^	0.99 ± 0.19^ac^	2.09 ± 0.50^cd^	1.02 ± 0.12^bc^
**Proximal jejunum**	0.84 ± 0.12^bd^	0.72 ± 0.10^c^	1.60 ± 0.38^cd^	0.93 ± 0.13^bc^
**Middle jejunum**	0.67 ± 0.08^cd^	0.87 ± 0.12^bc^	0.73 ± 0.14^d^	1.30 ± 0.22^bc^
**Ileum**	1.92 ± 0.28^a^	1.13 ± 0.20^ac^	2.66 ± 0.64^bd^	1.01 ± 0.17^bc^
**Caecum**	0.48 ± 0.12^cd^	1.99 ± 0.34^ab^	0.91 ± 0.29^d^	1.39 ± 0.20^b^
**Proximal colon**	0.83 ± 0.12^bd^	1.09 ± 0.16^ac^	1.74 ± 0.35^cd^	1.09 ± 0.18^bc^
**Distal colon**	1.01 ± 0.14^bc^	2.01 ± 0.35^a^	2.07 ± 0.42^cd^	0.87 ± 0.17^bc^
***P*value**	<0.001	<0.001	<0.001	<0.001

^a–d^Protein expressions within one column are different at *P* < 0.05 if they do not share a common letter.

Protein expression of B^0^AT1 was highest in the ileum and gastric cardia and lowest in the gastric fundus. Protein expression of ASCT2 was highest in parts of the large intestine, with lowest levels in all the three gastric regions and proximal jejunum. Protein expression of LAT2 was remarkably high in the oral mucosa and gastric cardia, whereas the pylorus, middle jejunum, and cecum had the lowest expression values. Protein expression of LAT4 was strikingly high in the oral mucosa with the lowest values in gastric fundus (Table 3).

**Fig. 4:**
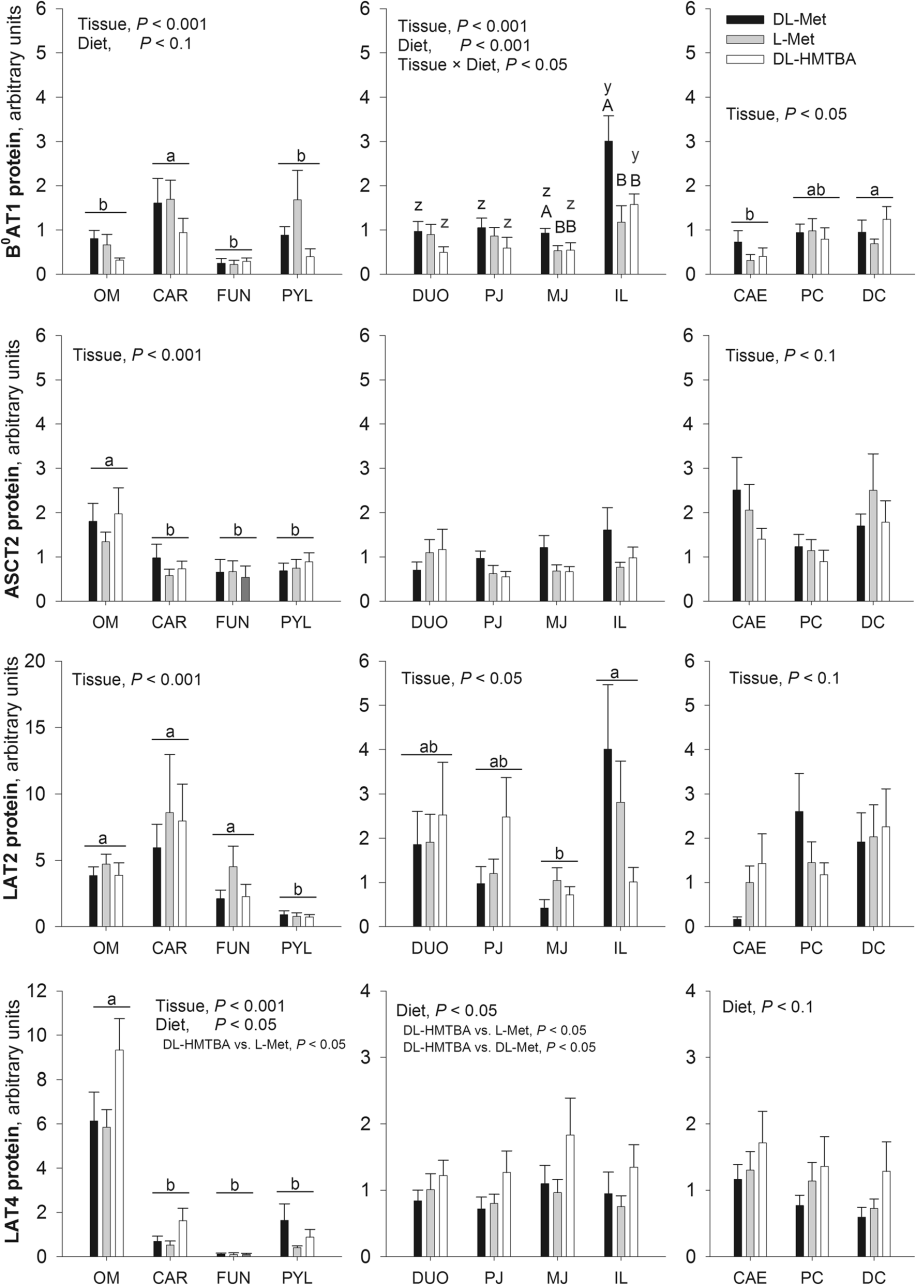
Western blot analysis of B^0^AT1, ASCT2, LAT2, and LAT4 in the intestinal and extraintestinal regions of pigs fed three different diets. Each value is the mean of 9 pigs and represents the relative expression to the IRC (IRC = 1). Data was compared with two-way ANOVA for the factors “Tissue”, “Diet”, and their interaction “Tissue × Diet”. Significant factor effects are mentioned in each graph. If column groups or columns do not share a common small letter within one graph, their expression values are either different irrespective of diet^(a–c)^ or within a given diet^(y,z)^ (*P* < 0.05). ^A,B^Different capital letters indicate diet effects within a given tissue. *OM*, oral mucosa; *CAR*, cardia; *FUN*, fundus; *PYL*, pylorus; *DUO*, duodenum; *PJ*, proximal jejunum; *MJ*, middle jejunum; *IL*, ileum; *CAE*, cecum; *PC*, proximal colon; *DC*, distal colon

The data were further divided into the three regional subgroups (similar to mRNA data) in order to investigate whether the diet has an effect on the protein expression in tissues of comparable physiological functions (Fig. 4). When comparing the expression of B^0^AT1 within each regional subgroup, protein levels in the small intestine were significantly affected by diet (*P* < 0.001) and tissue (*P* < 0.001) with significant interaction of the two factors (*P* < 0.05). The basis for interaction were higher B^0^AT1 protein levels in pigs fed DL-Met compared with L-Met or DL-HMTBA in the middle jejunum and ileum only (*P* < 0.05). In the extraintestinal regions, pigs fed DL-Met or L-Met tended to show higher B^0^AT1 protein levels when compared with DL-HMTBA (*P* < 0.1; Fig. 4). Among the large intestinal regions, two-way ANOVA identified higher B^0^AT1 expression in distal colon than the cecum—an effect that had not been significant with the all-tissue ANCOVA.

No diet effects were identified for ASCT2 protein (Fig. 4), which was unexpected because feeding a diet containing DL-Met had induced higher ASCT2 mRNA abundance in small intestinal segments (Fig. 2). Nonetheless, when only the proximal jejunum, middle jejunum, and ileum were tested in a two-way ANOVA, the effect of diet on ASCT2 protein was significant (*P* < 0.01) with higher expression in DL-Met–fed pigs compared with the other two groups.

No diet effects were observed for protein expression of LAT2 (Fig. 4). However, an effect of diet was observed for LAT4 in extraintestinal regions and small intestine (*P* < 0.05) and, as a trend also in the large intestine (*P* < 0.1). Pigs fed DL-HMTBA showed or tended to show highest LAT4 protein levels compared with pigs fed L-Met (*P* < 0.05 in extraintestinal and small intestinal tissues) and partly DL-Met (*P* < 0.05 in small intestinal tissues; Fig. 4).

## Discussion

The present study intended to investigate the longitudinal heterogeneity of the expression of AA transporters along the GIT of pigs and to elucidate a possible impact of Met supplementation on the expression of these transporters. The effect of targeted Met supplementation on the expression of gastrointestinal Met transporters has not been investigated previously, except for a study in broiler chicken where a trend for higher mRNA expression of *ATB*^*0,+*^ and *B*^*0*^*AT1* was observed in the ileum of L-Met– and DL-Met–supplemented versus non-supplemented control chickens [23]. Upon supplementation of a DL-Met–containing diet, we had observed an increased absorption of L-Met in the small intestinal segments (duodenum, middle jejunum, and ileum) and the induction of Na-dependent L-Met absorption in the middle jejunum for the same pigs used in the present study [14]. Therefore, the effects of the DL-Met–containing diet on transporter expression were of special interest.

Regarding the longitudinal distribution of Met transporters, it may appear somehow surprising that all investigated transporters were detectable in all investigated segments despite rather different physiological functions of these segments. The accepted textbook knowledge is that primarily the small intestine has relevance for AA absorption in mammals [24–27]. It has been shown specifically in pigs that their colon is also able to actively transport Met immediately after birth; however, this transport capability fades away in the first 10 days of life [28]. Nonetheless, several experimental findings provide indirect hints for a possible capacity to absorb AA also from the large intestine of adult mammals [29]. These hints include the appearance of microbial-derived AA nitrogen in the circulation and metabolism of pigs, humans and other non-ruminant mammals [30, 31], and the preferential presence of certain apical AA transporters like ATB^0,+^ [32, 33] and ASCT2 [34] in the large intestine of mice. As these transporters accept D-AA as substrates [32, 35], it may be assumed that they have a specific role in the recovery of D-AA from bacterial metabolism [32]. Nonetheless, a final proof for quantitatively relevant absorption of AA from the large intestine is still missing in mammals, including pigs and humans. Similarly, a final proof for the relevance of apically located AA transporters in stomach and oral mucosa is missing, although the presence of all Na^+^-dependent apical Met transporters investigated in the current study (B^0^AT1, ATB^0,+^, IMINO, and ASCT2) had been demonstrated in stomach, at least at the mRNA level, already in previous studies [14].

Coherent with a primary role of the small intestine in AA absorption, expression of *B*^*0*^*AT1* and *rBAT* mRNA was rather low in most extraintestinal and large intestinal tissues in the present study. However, partly coherent with the just cited literature findings, *ASCT2* mRNA was highest in gastric fundus and pylorus among all investigated tissues, *IMINO* mRNA showed values in the cardia, pylorus, and large intestine that were comparable to those of the small intestinal segments, and *ATB*^*0,+*^ mRNA was highest in the large intestinal segments, gastric cardia, and pylorus. On the protein level, B^0^AT1 was highest in the ileum; however, the cardia also showed a comparably high expression. For ASCT2 protein, highest values were observed in the oral mucosa, cecum, and distal colon.

The Na^+^-dependent carrier B^0^AT1 has previously been termed the Met-preferring system and is postulated to be a main carrier for Met absorption from the GIT of mammals [14]. Supporting its major role in AA absorption, we found transcripts predominantly in the small intestine with highest levels in the middle jejunum. Although *B*^*0*^*AT1* mRNA levels were not influenced by the feeding regimen, B^0^AT1 protein levels were regulated post-transcriptionally by the diet with a tissue × diet interaction in the small intestine. Pigs fed DL-Met showed a stronger B^0^AT1 protein expression in the middle jejunum and ileum. This is in partial support of a functional induction of a Na^+^-dependent transporter in the middle jejunum observed in our recent study on tissues of the same animals [22] and also partly confirms the previous results obtained in chickens receiving an L-Met– or DL-Met–supplemented diet [23]. It may thus be speculated that the increased expression of B^0^AT1 protein upon feeding a DL-Met–containing diet may have functional significance.

ATB^0,+^ has repeatedly been cited as a very important Met carrier in several species [36, 37]. Its expression in pigs is subject to controversy because a previous study was unable to detect ATB^0,+^ on a functional level in the porcine jejunum [38, 39]. Nonetheless, another study observed *ATB*^*0,+*^ mRNA expression in the small intestine and stomach of White Duroc × Chinese Erhualian pigs [40]. In our study, the levels of *ATB*^*0,+*^ mRNA were comparatively low in the small intestine (except for the duodenum) when compared with areas in the stomach and especially to the large intestine. This partly confirms another study in mice where ATB^0,+^ protein was predominantly expressed in the large intestine and, to a lower extent, also in the distal parts of the small intestine. The authors speculated that transport of Met and other AA into the intestinal epithelial cells might not be the primary function of ATB^0,+^ as most AA coming from feed intake are not present in the digesta this far in the intestinal canal [32]. As stated earlier, however, other scientists considered a functional relevance of transporters like ATB^0,+^ for large intestinal absorption of amino acid derived from microbial metabolism [29–31]. Dietary upregulation of *ATB*^*0,+*^ mRNA by a DL-Met or L-Met–containing diet, as previously observed in chicken [23], could not be identified in the present study. However, a trend for diet effect in the large intestine mRNA data indicates a possibility of dietary upregulation of ATB^0,+^ by DL-HMTBA supplementation in the large intestinal segments. This finding appears concordant with the work of Malik et al. who showed that DL-HMTBA is available in the digesta further down the lower intestine compared with Met [41]. Thus, DL-HMTBA is still present in the lumen of the large intestine and its possible microbial conversion to Met could potentially be associated with an upregulation of *ATB*^*0,+*^ in this portion of the gastrointestinal tract.

The Na^+^-independent transporter b^0,+^/rBAT has been postulated as a main uptake system for L-Met in Caco2 cells [42]. As mentioned earlier, *rBAT* mRNA expression was analyzed representatively for the expression of the b^0,+^/rBAT heterodimer in the present study. Transcripts of *rBAT* were also expressed highest in the small intestine, similar to *B*^*0*^*AT1*, suggesting the possibility of functional relevance in these tissue segments. In the proximal jejunum, mRNA expression of *rBAT* was increased by a diet containing DL-HMTBA; however, no difference was observed in other segments of the small intestine. Furthermore, as we did not observe increased Na^+^-independent methionine transport in the duodenum after supplementation of a DL-HMTBA–supplemented diet in our previous study [22], the functional significance of this finding remains to be determined.

The IMINO and ASCT systems have been characterized as uptake systems with low affinity for L-Met [34, 42]. They have been described as strongly expressed in the small intestine [14]. In the present study, the transcript levels of *IMINO* and *ASCT2* were rather similar in the small and large intestines. Additionally, ASCT2 protein levels showed only moderate variation across small and large intestinal segments with highest levels in the cecum and distal colon. Of note, a diet effect was observed for *ASCT2* mRNA in all small intestinal segments with highest values observed in pigs receiving the DL-Met–containing diet. Although the ASCT2 protein pattern almost mirrored its mRNA pattern in the proximal jejunum, middle jejunum and ileum, this effect did not penetrate towards statistical significance at the protein level when tested for all four small intestinal segments. When tested for only the proximal jejunum, middle jejunum and ileum, however, a DL-Met–containing diet significantly upregulated ASCT2 protein expression, which was coherent with the observed changes in mRNA expression. It was further coherent with the induction of a Na^+^-dependent transporter by the DL-Met diet observed in the middle jejunum of these pigs in our companion study [22].

The basolateral exit of Met is mainly mediated by a single uniport system, LAT4. The other transport systems known to accept Met on the basolateral side of enterocytes either operate as exchange proteins (LAT1, LAT2, y^+^LAT1) or mediate Na^+^-dependent basolateral import of Met from blood (SNAT1, SNAT2) [14]. In the present study, we evaluated transcripts (*y*^*+*^*LAT1*, *LAT2*, *LAT4*, and *SNAT2*) and proteins (LAT2 and LAT4) of basolateral transporters involved in Met shuttle. It needs to be acknowledged that y^+^LAT1 and LAT2 have been suggested to have a function in methionine reentry from the blood into the intestinal epithelial cell [14, 43]. LAT1 and LAT2 transporters do not contribute to a net flux of AA since they only exchange abundant AA for less abundant AA [19]. Because of this characteristic, it is not surprising that LAT2 is also expressed in cells that have no absorptive function like Paneth’s cells [44]. Transporter y^+^LAT1 exchanges intracellular cationic AA against extracellular neutral AA like Met. Only LAT4 transports selected AA (L-Met, L-leucine, L-isoleucine, and L-phenylalanine) solely by concentration gradient, functioning as a symmetrical uniporter [45, 46].

The expression of mRNA or protein of basolateral Met transporters was not increased by dietary supplementation with either DL-Met or L-Met. However, DL-HMTBA supplementation enhanced gene expression of *y*^*+*^*LAT1* in large intestinal tissues and LAT2 in small intestinal tissues, as well as protein expression of LAT4 in small intestinal tissues, extraintestinal tissues, and, as a trend, in large intestinal tissues. A similar stimulating effect of DL-HMTBA on basolateral Met transport was suggested in a previous study in chickens [47]. Of note, the increased expression of basolateral transport systems was apparently not associated with increased transepithelial Met absorption as the latter was not stimulated by HMTBA in our previous study [22]. This argues against a rate-limiting role of basolateral Met transporters for transepithelial Met absorption as postulated earlier [14] based on studies in Caco2 cells [48]. Another study showed that even with feeding DL-HMTBA, the first pass utilization of Met remains at a constant proportion of about 30% [49, 50], indicating that basolateral transporters do probably not create an intracellular trap for Met in pigs as long as dietary Met concentrations are within requirement ranges.

Methionine metabolism in enterocytes plays a substantial role in the gastrointestinal absorption of Met. It is estimated that 20–30% of dietary Met is directly metabolized in intestinal epithelial cells [49, 50]. Dietary supplementation of DL-HMTBA is often seen as a way to avoid this first pass metabolism because HMTBA passes the intestinal epithelial cell largely unchanged and is only later converted into Met in the liver [51, 52]. However, such interpretation does not hold true. At least for pigs, HMTBA is absorbed from the intestinal lumen more slowly than L-Met and, thus, has a greater loss to intestinal bacterial degradation (as part of the first pass metabolism), associated with a lower amount of HMTBA absorption [41]. The latter may explain lower plasma Met concentrations by dietary HMTBA supplementation compared with Met supplementation [53]. Thus, it may be speculated that dietary DL-HMTBA supplementation induces a higher expression of basolateral Met “recovery systems” in order to compensate the lower dietary Met levels and to enhance the exchange of Met with other AA.

An interesting finding of the present study was that mRNA expression of two transporters, namely B^0^AT1 and LAT2, was higher in the middle jejunum compared with the ileum, whereas their protein expression pattern was inverse. Bringing this data together with our previous flux study, it conformed functionally with higher absorptive capacity for L-Met in the ileum compared with the middle jejunum, at least at higher L-Met concentrations [22]. A quite similar finding was reported earlier for the glucose transporter GLUT2. Despite lower mRNA levels in the distal ileum compared with the middle jejunum, GLUT2 protein abundance was higher in the distal ileum than the middle jejunum [54]. Considering these findings together, it is tempting to suggest that a higher absorptive capacity for nutrients in the ileum of pigs compared with their middle jejunum originates partly from differences in the translation or turnover of certain nutrient transporters.

The present study was performed in pigs; however, Met supplements gain increasing popularity also in human nutrition [14]. As humans rely on L-Met supplements almost exclusively, it is desirable to explore in model animals whether other Met supplements may have nutritional benefits beyond those of L-Met. Pigs are an ideal model for humans [55]. Despite some minor differences, the anatomical and physiological similarities between the porcine and human intestines are striking and far superior when compared with rodent models or other non-rodent species [56]. Gut microbiota and nutrient digestibility of pigs show great resemblance to the human intestine [57]. The high correlation of ileal amino acid digestibility and the similarity in eating habits between pigs and humans make pigs a useful model for, especially, protein digestion in humans [58]. As such, the present results have a high potential to be transferable to man, suggesting that the type of Met supplementation may affect transporter expression and absorptive efficiency for Met and potentially other amino acids.

## Conclusion

The present study showed that known Met transporters have a distinct longitudinal pattern of expression along the different sections of the GIT in pigs. A high expression of several Met transporters in small intestinal segments underlines the primary role of these segments in AA absorption. However, some transporters showed rather high expression in segments that do not have a proven role in AA absorption (extraintestinal and large intestinal tissues). Dietary Met source changed the expression of some transport systems. From these changes, it may be extrapolated that a diet containing DL-Met has potential to increase apical Met transport in the small intestine, which is congruent with recent functional findings of our group. On the other hand, a diet containing DL-HMTBA has potential to increase basolateral Met transporters in the small intestine and, partly, other gastrointestinal tissues. However, this was not previously found to improve functional methionine absorption and may be a result of a system compensation for lower free Met to be used for epithelial cell metabolism or as an exchange molecule for transport of other AA. Overall, the degree of regulation appeared small to moderate and, likely attributable to this fact, changes in mRNA expression did not clearly correlate with changes in protein expression. Importantly, the small changes in mRNA (ASCT2) and protein expression (B^0^AT1) of apical Na^+^-dependent transporters in the present study cannot explain the *de novo* induction of a Na^+^-dependent uptake system in the mid jejunum identified in our previous study upon feeding a DL-Met–containing diet [22]. Therefore, further functional studies on intestinal Met absorption should complement the current findings, including investigations on posttranslational mechanisms that possibly regulate AA absorption. A comprehensive knowledge on transcriptional, translational, and posttranslational regulation of AA absorption will greatly enhance our understanding of AA absorption in animals and eventually humans.

## Data Availability

The datasets used and/or analyzed during the current study are available from the corresponding author on reasonable request.
